# Exploring the role of traditional Chinese medicine rehabilitation in stroke based on microRNA-mediated pyroptosis: A review

**DOI:** 10.1097/MD.0000000000039685

**Published:** 2024-09-20

**Authors:** Hanwen Ma, Luwen Zhu

**Affiliations:** a Graduate School, Heilongjiang University of Traditional Chinese Medicine, Harbin, China; b Rehabilitation Center, The Second Affiliated Hospital of Heilongjiang University of Chinese Medicine, Harbin, China.

**Keywords:** microRNA, pyroptosis, review, stroke, traditional Chinese medicine rehabilitation

## Abstract

Stroke, also known as “cerebrovascular accident,” is a disease caused by acute impairment of brain circulation, which has a high rate of disability and mortality. Ischemic stroke (IS) is the most common type of stroke and a major cause of death and disability worldwide. At present, there are still many limitations in the treatment of IS, so it may be urgent to explore more treatments for IS. In recent years, the clinical application of traditional Chinese medicine rehabilitation methods such as traditional Chinese medicine, acupuncture, massage, traditional exercises and modern rehabilitation technology has achieved good results in the treatment of IS. Concurrently, studies have identified microRNA (miRNA), which are intimately associated with traditional Chinese medicine rehabilitation, as regulators of pyroptosis through their influence on microglia activity, inflammatory response, oxidative stress, angiogenesis and other factors, but at present, the mechanism of this direction has not been systematically summarized. Consequently, this article delineates in detail the specific role of miRNA in IS and the related activation pathways of pyroptosis in IS. This article presents a detailed discussion of the role of microRNA-mediated pyroptosis in IS, with a particular focus on the signaling pathways involved. The aim is to provide new insights for the research of traditional Chinese medicine (TCM) rehabilitation in the prevention and treatment of IS. In addition, the article explores the potential of TCM rehabilitation in regulating miRNA-mediated pyroptosis to intervene in IS.

## 1. Introduction

Stroke, also known as “cerebrovascular accident,” refers to the obstruction of cerebral arteries or the sudden rupture of blood vessels that impede blood circulation in the brain. This can be further categorized into 2 main types: ischemic and hemorrhagic. IS is the most common type of stroke, which is a clinical syndrome that causes local brain tissue ischemia and hypoxic necrosis due to cerebrovascular diseases, which quickly leads to corresponding nerve function deficit, accounting for 87% of stroke cases.^[1]^Over the past 2 decades (1990–2019), the absolute number of strokes occurred increased by 70%, the absolute number of strokes affected increased by 85%, the number of stroke deaths increased by 43%, and the disability adjusted life years increased by 32%. It is important to further study the mechanism of IS and explore more possible treatment approaches.^[1]^

miRNA is a small non-coding RNA molecule that regulates gene expression. Through base pairing with complementary sequences of the 3 ‘-non-translation region of the target messenger RNA(mRNA), miRNA can negatively mediate gene expression at the post-transcriptional level, leading to mRNA degradation or translation inhibition. Currently, it IS considered to be a specific diagnostic indicator of IS, which also plays an important role in neurodevelopment and neuronal function.^[2]^ After IS, miRNA can regulate the expression of genes related to inflammation, cell communication, neuroprotection and recovery.^[3]^ This brings hope for effective prevention, diagnosis and treatment of stroke and stroke-related complications. Pyroptosis, a recently identified pro-inflammatory form of regulated cell death, is a programmed cell death associated with inflammation.^[4]^ It is characterized by the formation of membrane pores mediated by the GasderminD protein (GSDMD), which causes rapid cell swelling and dissolution, resulting in the release of large quantities of pro-inflammatory mediators. This process not only causes local inflammation but can also lead to the amplification of inflammation and is closely associated with cardiovascular disease.^[5]^ In normal physiology, cell pyroptosis plays an important role in the defence against pathogen infection. However, excessive pyroptosis may result in excessive and persistent inflammation, which can lead to inflammatory disease.^[6]^

Over the past 2 decades, stroke therapy can be divided into activity-based therapy, drug therapy, and cell therapy.^[7]^ Activity-based therapies aim to accelerate the recovery of motor function after IS, including robot-assisted upper limb training after stroke,^[8]^ early speech rehabilitation,^[9]^ etc. However, there are still few specific studies on this aspect. Drug-based therapies include tissue plasminogen activators, gamma-aminobutyric acid receptor agonists, etc. Among them, the former has a limited therapeutic window, may be of little help to neurological recovery,^[10]^ and lacks future prevention, while the latter, despite positive results in animal models, has not yet been observed for complete treatment. Cell-based therapies include stem cell therapy, nerve stem therapy, etc. However, studies have found that the efficiency of stem cell therapy is significantly affected by specific types of stem cells,^[11]^ and the efficacy of exogenous stem cells cannot be definitively guaranteed. Currently, many drug - and cell-based therapies have been used in a variety of in vitro and in vivo studies, but with varying success rates and limitations.^[12]^ Although society’s understanding of stroke pathophysiology has increased over the past decade due to the high prevalence of stroke and the increase in stroke-related deaths, insights into stroke recovery remain limited. Previously, TCM rehabilitation treatment methods based on acupuncture, massage, traditional Chinese medicine, traditional sports and related modern treatment techniques have played their potential in clinical practice, with advantages of low side effects, high safety and good therapeutic effect.^[13,14]^

After IS, a large number of inflammatory factors are released, leading to pyroptosis and thus aggravating the local inflammatory response. miRNA can regulate inflammation and affect nerve function. This article will therefore commence with an overview of the 3 key areas of miRNA, pyroptosis and TCM rehabilitation, before summarizing the related effects and research progress of TCM rehabilitation on IS, with a particular focus on miRNA-mediated pyroptosis. This will provide a theoretical basis for further research on the pathogenesis and treatment of IS, as well as offering innovative treatment ideas.

## 2. Search strategy and selection criteria

We conducted comprehensive searches of PubMed, Cochrane Library, Embase and Web of Science for literature published in English up to August, 2024, using a specific set of search terms: “Stroke” AND “microRNA” AND “Pyroptosis” AND “Acupuncture” OR “Tuina” OR “Traditional achievement method” OR “Modern rehabilitation techniques.” We tailored this search strategy to suit the indexing styles of the different electronic databases. We selected articles that reported on diagnostics, pathophysiology, or mechanisms, and focused on the intervention and the outcomes. We also considered publications cited in these articles that did not appear in the search algorithm. If the previously selected articles cited a respective article frequently. The final selection of cited articles reflects our subjective assessment of their relevance with respect to the reported results and the methodological quality of the reported work.

## 3. The role of miRNA in IS

### 3.1. miRNA and IS

**Figure 1.**
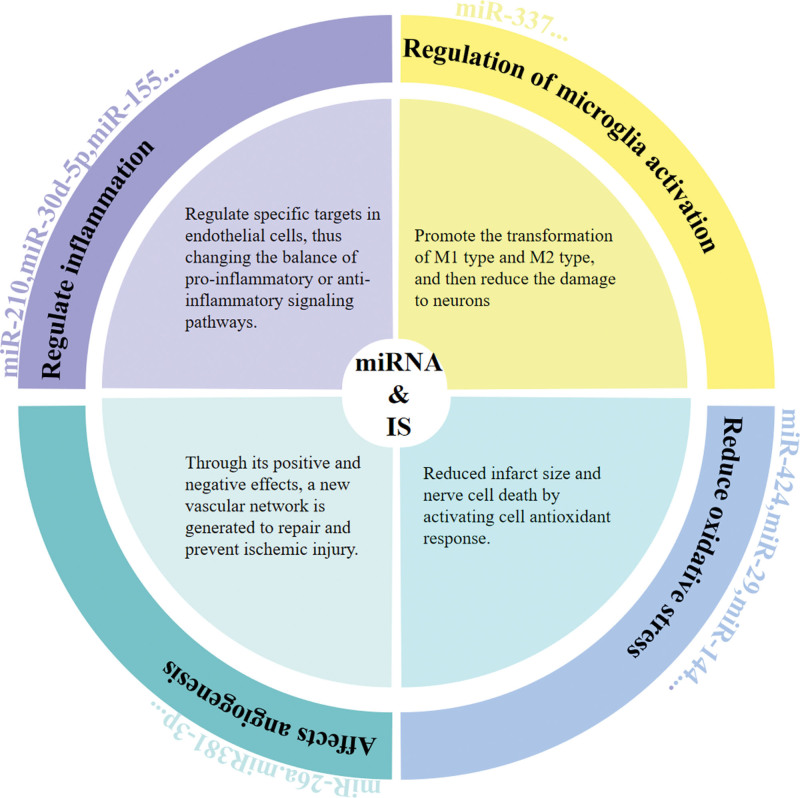
The role of miRNA in stroke. IS = ischemic stroke, miRNA = microRNA.

miRNA is a kind of small non-coding RNA molecule that regulates gene expression. They can negatively regulate gene expression at the post-transcriptional level by base pairing with complementary sequences in the 3’-untranslated region of target mRNA, which leads to mRNA degradation or translational repression. Currently, it is considered that miRNA can be used as a specific diagnostic index (Fig. 1). Furthermore, it is evident that miRNA plays an important role in neural development and the function of neurons.^[2]^ Following ischemia-hypoxia and reperfusion injury in brain tissue, a large number of endogenous danger signals and related inflammatory mediators will be produced, thereby activating inflammasomes and inducing pyroptosis. This will result in the release of a large number of pro-inflammatory cytokines. miRNA can regulate the inflammatory response directly through its related targets, and further affect the inflammatory process by regulating the activity of microglia, thus affecting the occurrence of pyroptosis after IS.^[3]^ Additionally, it can affect angiogenesis and accelerate the prognosis of IS. This offers a promising avenue for the effective prevention, diagnosis and treatment of stroke and stroke-related complications.

### 3.2. Regulation of inflammatory response

Following an acute stroke, the degree of neuronal cell death may vary depending on the severity of the ischemia and the energy expenditure. This may result in the triggering of secondary neuroinflammation, which may deepen the tissue damage.^[15]^ Consequently, an investigation into the efficacious pathways of miRNA regulation of the inflammatory response may result in a reduction of damage and an improvement in the prognosis of patients with IS. Huang et al demonstrated that the inhibition of miR-210 suppressed proinflammatory responses and reduced brain damage in an acute phase mouse model of IS.^[16]^ In response to biochemical and biomechanical stimuli, miRNAs regulate specific targets in endothelial cells, thereby altering the balance of proinflammatory or anti-inflammatory signaling pathways.^[17]^ Jiang et al found that IS patients and animal models exhibited increased expression of inflammatory cytokines and decreased expression of anti-inflammatory cytokines interleukin-4 (IL-4), interleukin-10 (IL-10) and miR-30d-5p. Furthermore, the study demonstrated that exosomes significantly reduced the infarct brain damage area by inhibiting autophagy and promoting M2 microglia/macrophage polarization in vivo.^[18]^ Yang et al conducted a study using the middle cerebral artery occlusion (MCAO) rat model, this study examines the commonly used prescription treatment for IS, the semen cassia twig decoction, and its potential role in inflammation. The results indicate that the semen cassia twig decoction, through miR-155, mediates nerve inflammation and generates a nerve protective effect, which helps to reduce cramps after ischemic stroke.^[19]^ Therefore, TCM rehabilitation method can effectively improve the related symptoms of IS through miRNA, but the relevant pathways and mechanisms mediated by miRNA are not clear, and further research is needed.

### 3.3. Regulation of microglia activation

Microglia are tissue-innate macrophages of the central nervous system that play an important role in monitoring and intervening in synaptic and neuronal activity.^[20]^ Microglia activation is a graded response in vivo that plays a pivotal role in safeguarding the nerve parenchyma from infectious diseases, inflammation, trauma, ischemia, brain tumors, and neurodegeneration.^[21]^ The regulation of microglia activation by miRNAs has a dual function. On the one hand, it positively regulates microglial activation. M1 microglia secrete a considerable number of pro-inflammatory cytokines, which can result in the destruction of nerve cells and the blood-brain barrier, as well as affecting neurogenesis.^[22]^ Consequently, the inhibition of microglial transformation to the M1 phenotype can result in a reduction in neuronal damage. Conversely, it also has a negative regulatory effect on microglial activation. The M2 phenotype is neuroprotective and can promote a reparative anti-inflammatory response. Consequently, it can effectively reduce neuronal damage and facilitate tissue repair by promoting the transformation of the M2 phenotype.^[23]^ Fan et al^[24]^ demonstrated that the knockdown of miR-377 could promote angiogenesis, inhibit the activation of microglia and the release of pro-inflammatory cytokines, thereby inhibiting brain inflammation and reducing cerebral infarction volume and ischemic brain damage of microglia in MCAO rats. At present, there are many studies on the regulation of miRNA on microglia, and further exploration of the typing of a single miRNA on microglia may promote the research progress of IS.

### 3.4. Reduce oxidative stress

The pathogenesis of neurodegenerative disease is primarily attributed to the apoptosis or necrosis of neurons, which subsequently leads to dysfunction and impaired cognitive function. Given its high metabolic rate and lipid content, the central nervous system is particularly susceptible to oxidative stress, which has attracted significant attention in the context of neurodegenerative diseases.^[25]^ In vivo studies have demonstrated that miR-424 overexpression protects against transient focal cerebral ischemia-reperfusion injury and inhibits hydrogen peroxide-induced oxidative stress injury, reducing cerebral infarct size and neuronal apoptosis. In vitro studies have shown that miR-424 overexpression activates cellular antioxidant responses, inhibiting oxidative stress and apoptosis in IS models.^[26]^ Ma et al^[27]^ treated an in vitro cell model of IS with miR-29 antamide for 48 hours and demonstrated that miR-29b was capable of inhibiting oxidative stress and apoptosis in IS and was significantly efficacious in the treatment of IS. Chu et al^[28]^ discovered that ginsenosides Rg1, by inhibiting the activity of miR-144, can promote Nrf2/ARE pathways of antioxidant defence, thereby reducing oxidative stress in IS. An increasing number of studies have identified miRNAs as potential targets and regulators of oxidative stress-related pathways. However, the specific pathways and mechanisms of miRNA remain to be elucidated.

### 3.5. Affects angiogenesis

The rupture or occlusion of cerebral vessels will lead to stroke, and the primary objective of angiogenesis following IS is to generate a new vascular network to repair and prevent ischemic damage. Given that IS is associated with a multitude of miRNA target genes, miRNA is regarded as a double-edged sword in cerebral angiogenesis.^[29]^ On the one hand, it exerts a positive regulatory effect. Liang et al^[30]^ established a model of middle cerebral artery occlusion in rats to induce permanent cerebral infarction, and found that miRNA-26a could promote angiogenesis in a rat model of cerebral ischemia through the PI3K/AKT and MAPK/ERK pathways. Furthermore, the potential role of miR-381-3p in MCAO rats and its underlying mechanism were evaluated, resulting in the determination that upregulation of miR-381-3p would inhibit the levels of Map3k8 and Cebpb, promote angiogenesis and reduce inflammation.^[31]^ Conversely, Sun et al^[32]^ demonstrated that the endothelial miR-15a/16-1 cluster acts as a negative regulatory factor in ischemic cerebrovascular disease, thereby impeding long-term neurological recovery. Furthermore, knockdown of miR-377 was observed to enhance angiogenesis and reduce cerebral infarct volume and brain damage.^[24]^ Given the multiplicity of miRNA effects on angiogenesis following IS, further investigation into the potential effects of miRNA in various contexts is warranted to provide novel insights for the treatment of stroke.

## 4. The role of pyroptosis in IS

### 4.1. Pyroptosis and IS

**Figure 2.**
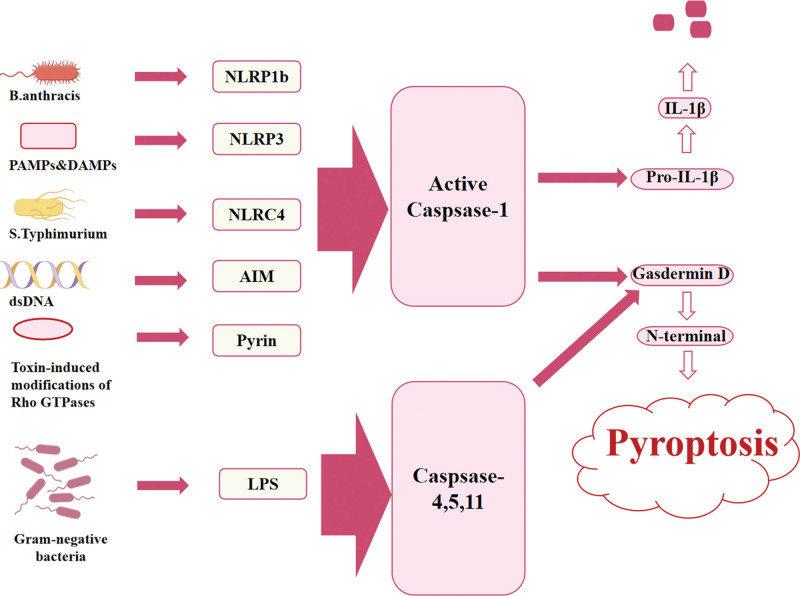
Overview of pyroptosis pathway. AIM = absent in melanoma, DAMP = damage-associated molecular pattern, IL = interleukin, LPS = lipopolysaccharide, NLRC = nucleotide-binding oligomerization domain like receptor subfamily C, NLRP = nucleotide-binding oligomerization domain-like receptor, PAMP = pathogen associated molecular pattern.

Both permanent ischemia and reperfusion after transient cerebral ischemia can induce brain tissue inflammation. Pyroptosis not only participates in the initiation of inflammatory response, but also plays a key role in disseminating inflammatory signals and amplifying inflammatory response.^[33]^ In addition, the inflammatory response after cerebral ischemia/reperfusion is more severe than that of persistent cerebral ischemia, and the development of pyroptosis and activation of its signaling pathway may also be more intense. The main inflammatory pathway in the early stage is Caspase/GSDMD, and the main pathway in the late stage is Caspase-3/GSDME (Fig. 2). Studies have demonstrated that miR-145 antagonists have neuroprotective effects in vivo after IS and can reduce focal brain injury by regulating miR-145/GSDMD-mediated pyroptosis.^[34]^ Meanwhile, by reducing the content of Caspase-1, it can further reduce the inflammatory response and the degree of pyroptosis after cerebral ischemia-reperfusion and play a neuroprotective role.^[35]^ In addition, it was observed that downregulation of miR-155-5p, which regulates the DUSP14/TXNIP pathway and subsequently modulates nucleotide-binding oligomerization domain-like receptor protein 3 (NLRP3), can ameliorate cerebral infarction in MCAO rats, suppress inflammatory response, and inhibit cell pyroptosis.^[36]^ At present, miRNAs have been identified as having the capacity to affect NF-κB-driven inflammatory pathways, reduce microglial pyroptosis, enhance white matter integrity, and improve relevant functional outcomes after IS.^[28]^ Consequently, the investigation of the mechanism of miRNA-mediated pyroptosis and inflammation may represent a promising avenue for the development of novel therapeutic strategies for IS.

### 4.2. Classical pyroptosis pathway

Caspase-1 is a cysteine protease, expressed in cells as an inactive precursor. The influence of Caspase 1 varies considerably depending on the cell type and the nature and size of the activation stimulus. In inflammatory and autoimmune diseases, for instance, Caspase-1 plays an important role in the pathogenesis of IL-1β and IL-18. It also fine-tunes cytokines in response to microbial stimulation, inhibits bacterial growth in cells, affects cell repair and survival, and more.^[37]^ The pyroptosis pathway, which is based on the Caspase-1 pathway, is caused by the formation of discrete pores in the plasma membrane, which promote the release of inflammatory factors, cell swelling, and osmotic lysis.^[38]^ Studies have shown that by inhibiting the expression of Caspase-1, it can inhibit pyroptosis, improve neurological function and reduce brain damage in the acute stage of ischemic stroke.^[39]^ In addition, the inhibition of Caspase-1 can inhibit the activation of pyroptosis and the RAGE/MAPK pathway, thereby significantly improving the permeability and integrity of the blood-brain barrier affected by ischemia.^[40]^ Gu et al^[41]^ demonstrated that activation of Raf kinase inhibitor proteins, in part through the ASC/Caspase-1/GSDMD pathway, could attenuate neuronal pyroptosis and brain injury after ICH. Zhang et al^[42]^ employed the down-regulated XBP-1 protein to suppress pyroptosis by inhibiting the NLRP3/Caspase-1/GSDMD pathway in the hippocampus, thereby mitigating the injury caused by cerebral ischemia-reperfusion.

### 4.3. Non-classical pyroptosis pathways

Similar to Caspase-1, non-canonical pyroptosis is mediated by Caspase-4/5/11, however, in contrast to Caspase-1, which is activated in the canonical inflammasome, Caspase-4/5/11 is activated in the non-canonical inflammasome through direct interaction with cytosolic lipopolysaccharide.^[43]^ Furthermore, activated Caspase-4/5/11 can cleave GSDMD into N-GSDMD, which oligomerizes and is transferred to the cell membrane, ultimately forming a plasma membrane pore.^[44]^ However, they were able to mediate IL-1β/IL-18 maturation and secretion through the NLRP3/Caspase-1 pathway in some cells.^[45]^ The discovery of Caspase-4/5/11 function extends the concept of pyroptosis mediators from Caspase-1 to the inflammations, thereby demonstrating that pyroptosis is not restricted to monocytes. Nevertheless, the role of Caspase-4/5/11 in cardiovascular disease is rarely reported, and this may be a future research direction in the cardiovascular field.

### 4.4. Other pyroptosis pathways

Caspase-8/9/10 can be initiated by the activation of apoptosis-related pathways, which are considered to be the executors of Caspase-3/6/7. Nevertheless, it has been demonstrated that Caspase-8 is also a molecular switch that controls pyroptosis, which can directly cleave GSDMD to induce pyroptosis.^[46]^ In addition, Caspase-3 has been demonstrated to induce pyroptosis by cleaving GSDME,^[47]^ while Caspase-6 has been shown to promote inflammasome activation, which in turn induces pyroptosis.^[26]^ Furthermore, some studies have identified ANXA3 as a potential pyroptosis-related gene marker in IS. ANXA3 has been demonstrated to inhibit pyroptosis through the NLRC4/AIM2 axis.^[48]^ The enzyme particle and lymphocyte-derived granzyme A have been demonstrated to induce Caspase-mediated cell pyroptosis.^[49]^ The former can be activated by Caspase-3 or directly cleaved by GSDME, while the latter can be directly cleaved by GSDMB, resulting in pyroptosis-induced cells. At present, research into miRNAs in pyroptosis is relatively limited, although it may offer new avenues for further investigation into the underlying mechanisms.

## 5. The methods of rehabilitation of TCM research progress in the IS

### 5.1. The TCM therapy

**Figure 3.**
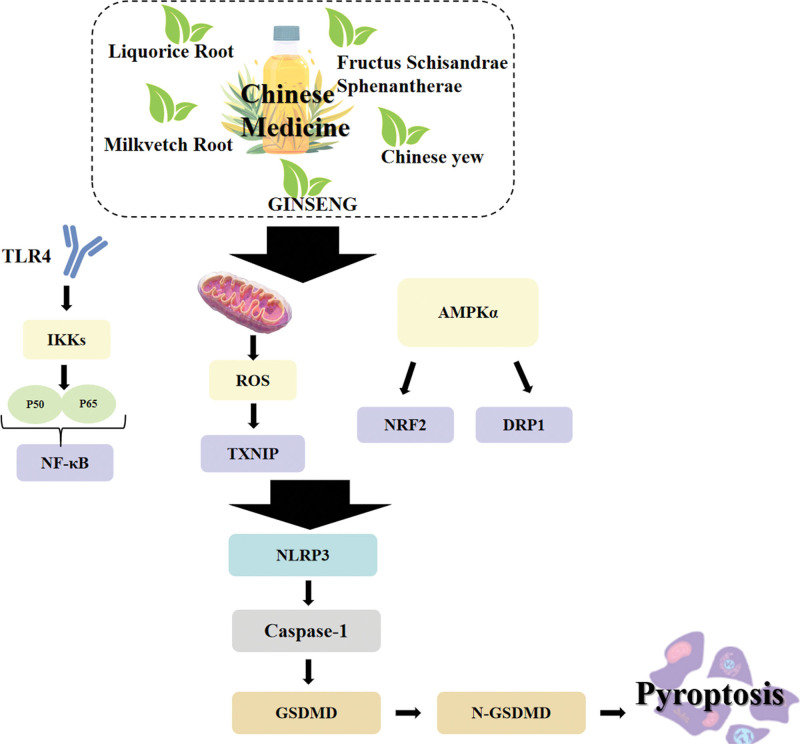
The mechanism of Chinese medicine therapy in cell pyroptosis. AMPK = AMP-activated protein kinase, DRP = dynamin-related protein, GSDMD = GasderminD protein, IKK = IkappaB kinase, NLRP = nucleotide-binding oligomerization domain-like receptor, NRF = NF-E2-related factor, ROS = mitochondrial reactive oxygen species, TXNIP = thioredoxin-interacting protein.

A growing body of research has focused on the potential therapeutic benefits of TCM in the context of stroke-induced pyroptosis. This encompasses the expression of related inflammasomes and classical pathways. Additionally, the impact of TCM on pyroptosis and anti-IS multi-pathways has been explored. The majority of TCM interventions aim to inhibit the activation of the NLRP3 inflammasome and Caspase-1 by regulating upstream signaling pathways. For instance, the TLR4/NF-κB, ROS/TXNIP, AMPK/Nrf2 or DRP1/NLRP3 signaling pathways can be employed to suppress cell pyroptosis and play a protective role in the context of IS (Fig. 3). Ginsenoside Rd has been demonstrated to up-regulate miR-139-5p, thereby attenuating pyroptosis mediated by the ROS/TXNIP/NLRP3 inflammasome axis.^[50]^ Gastrodin has been demonstrated to down-regulate NLRP3, Caspase-1 and related inflammatory factors by regulating the long non-coding RNA NEAT1/miR-22-3p axis, ultimately inhibiting pyroptosis and reducing IS injury.^[51]^ Furthermore, the TCM compound TaohongSiwu Decoction has been demonstrated to inhibit the activation of the NLRP3 inflammasome by inhibiting the HMGB1/TLR4/NFκB and MAPK signaling pathways, down-regulate GSDMD, and inhibit pyroptosis in IS rats.^[52]^ The advantages of TCM in the prevention and treatment of IS are evident in terms of early treatment and global coordination. Over the past few decades, the efficacy of TCM and its bioactive components has been scientifically demonstrated.^[53]^ The regulation of pyroptosis by TCM has emerged as a novel research focus in the field of stroke-induced brain injury intervention.

### 5.2. Acupuncture therapy

Acupuncture has been employed for millennia in the treatment of stroke patients and those recovering from stroke. Studies have demonstrated that acupuncture can facilitate nerve growth in individuals with IS.^[32]^ Cupping thrombolysis treatment of cerebral infarction, acupuncture of early intervention has been shown to inhibit the cerebral infarction in the rat brain cortex by suppressing the excessive expression of Caspase-3, significantly improving the ultrastructure of neurons, and to a certain extent, enhancing the therapeutic effect of thrombolysis.^[54]^ Acupuncture at “Baihui” (GV20) and “Shenting” (GV24) has been demonstrated to significantly reduce the percentage of cerebral infarction volume, neuronal apoptosis rate and microglia activation in the rats with cerebral ischemia-reperfusion injury. Additionally, electroacupuncture has been shown to alleviate cerebral ischemia-reperfusion injury in rats by regulating melatonin-NLRP3 to inhibit pyroptosis.^[55]^ The efficacy of acupuncture has been substantiated by numerous studies. However, the precise mechanism by which acupuncture therapy exerts its effects on miRNAs and cell pyroptosis remains to be elucidated.

### 5.3. The massage therapy

The study found that after massage treatment at Baihui, Hegu, Yanglingquan, Zusanli, Sanyinjiao and Taichong and other acupoints, such as cell Caspase-1 pyroptosis-related molecules, IL-1β, IL-18 and GSDMD expression all down, can improve impaired neurocognitive function by inhibiting NLRP3-induced pyroptosis.^[56]^ By intervening the key acupoints on the gallbladder meridian of foot shaoyang, massage manipulation can effectively down-regulate miR-21 and regulate the expression of related proteins and factors in the upstream and downstream of the TLR8/ERK pathway, thus reducing the inflammatory response at the pain site, while miR-21 has been confirmed to regulate NLRP3 inflammation-mediated IL-1β secretion and Caspase-1 activation to inhibit cell pyroptosis.^[57]^ Studies have demonstrated that TLR4 can facilitate DDX3X-mediated NLRP3 inflammasome activation via the JAK2/STAT1 pathway, thereby exacerbating microglial pyroptosis.^[58]^ Tuina therapy has been shown to inhibit peripheral inflammation by regulating TLR4 pathway and miRNA, regulating channels, inhibiting the activation of glial cells, regulating brain function, and play an analgesic role.^[59]^ Nevertheless, the precise mechanism by which tuina therapy regulates pyroptosis through miRNA remains poorly understood, and numerous additional mechanisms warrant further investigation.

### 5.4. The traditional achievement method

It has been demonstrated that participation in sports can promote the generation of neurotrophic factor, or reduce vascular risk factors. The traditional exercises combine a number of elements, including ideas, breathing, posture and other aspects, which can effectively relax the patient and improve their limb function and balance ability. Furthermore, these exercises can improve and promote the plasticity of neurons. Studies have demonstrated that Tai Chi can enhance skeletal muscle function in the elderly through the TNF-α/Caspase-8 pathway^[60]^ Caspase-8 can also influence the process of pyroptosis. Furthermore, Tai Chi exercise up-regulates brain-derived neurotrophic factor (BDNF), which has been demonstrated to repair damaged nerves and promote nerve regeneration and functional recovery.^[61]^ BDNF can inhibit the formation of NLRP3 inflammasome and pyroptosis through glucose metabolism regulation and mitochondrial homeostasis protection.^[62]^ Including qigong, tai chi, meditation and mindfulness therapies such as yoga, can through increase of telomerase activity, thereby delaying the aging process, reducing oxidative stress, enhancing resistance to apoptosis, promoting neurotrophic factors and facilitating nerve regeneration. Concurrently, it can also inhibit the stress and nerve inflammation induced by NF-κB, regulate gene expression and DNA methylation, and prevent the progression of neurodegenerative diseases.^[63]^ Furthermore, traditional exercises such as Baduanjin and Wuqinxi also play an important role in the treatment of IS, However, there are few studies on their correlation with pyroptosis, which may provide a new direction for the research of traditional exercises in the treatment of stroke.

### 5.5. Modern rehabilitation techniques

Previous studies have indicated that modern rehabilitation techniques such as transcranial magnetic stimulation and hyperbaric oxygen therapy are associated with pyroptosis. It has been demonstrated that repetitive transcranial magnetic stimulation can reduce motor dysfunction and neuronal pyroptosis caused by cerebral ischemia-reperfusion injury by inhibiting the expression of neuronal pyroptosis-related proteins in the peri-infarct area.^[64]^ Studies have demonstrated that the combination of transcranial magnetic stimulation and acupuncture can inhibit the NLRP3 inflammasome, which is associated with pyroptosis and the production of inflammatory factors, Caspase-1 and IL-1β.This results in a reduction in the inflammatory immune response and the alleviation of pain in patients.^[65]^ Ye et al^[66]^ demonstrated that hyperbaric oxygen therapy inhibited neural stem cell pyroptosis by inhibiting the long non-coding RNA-H19/miR-423-5p/NLRP3 axis, and that the inhibition of lncrNA-H19-related pyroptosis by hyperbaric oxygen therapy favored neural stem cell proliferation and neuronal differentiation. Furthermore, hyperbaric oxygen therapy was found to be effective in improving the production of pyroptosis-related proteins, including IL-1β and IL-18 by regulating NLRP3 inflammasome activation and Caspase-1.^[67]^ The needle kang method represents a more effective approach to the treatment of stroke, with the combined use of new treatments gradually demonstrating its advantages. Consequently, in the context of miRNA-mediated pyroptosis, the potential role of traditional therapy in conjunction with novel rehabilitation technology in the treatment of IS warrants further investigation.

## 6. Summary

**Figure 4.**
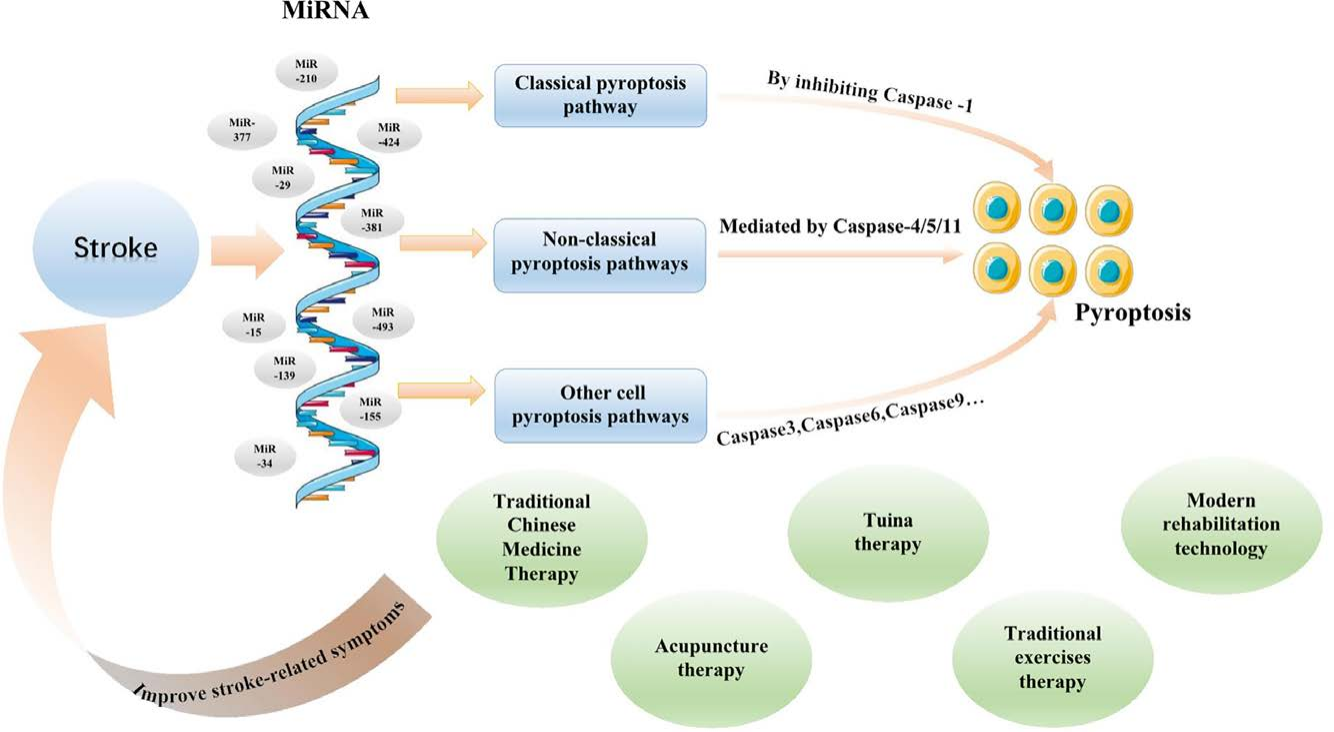
Traditional Chinese medicine rehabilitation approaches improve stroke-related symptoms pathways by regulating miRNA-mediated pyroptosis. miRNA = microRNA.

The occurrence of stroke is often the result of a variety of pathogenic factors, and its pathogenesis is complex, with symptoms that change rapidly. According to TCM, the causes of IS are wind, fire, phlegm, qi, blood stasis and deficiency, and the main treatment principles include syndrome differentiation and treatment, 3 causes, etc. TCM treatment of IS has been proved to be effective and practical. miRNAs has great potential as biomarkers for IS diagnosis, prognosis and brain injury, and play an important role in pyroptosis by mediating the expression of key proteins (Fig. 4). At the same time, pyroptosis has also become a potential therapeutic target to prevent excessive cell death in IS.^[43]^ Consequently, in the various stages of the disease, miRNA-mediated cell pyroptosis can be employed as a prognostic, diagnostic, protective, and therapeutic approach. TCM rehabilitation therapy encompasses TCM, acupuncture, massage, traditional exercises and modern rehabilitation techniques. These can regulate pyroptosis through miRNA and affect the process of disease, thereby reducing brain tissue damage after IS (Table 1). Further clarification is required regarding the target and related mechanisms of miRNA-mediated cell pyroptosis treatment in the context of IS. Currently, the focal death pathways associated with IS are limited to the classic pathways, such as Caspase-3 mediated pyroptosis. The other way mediated pyroptosis, which is distinct from the classic pathways, still requires further research to be fully elucidated. In addition, the literature on the rehabilitation of TCM regulation of miRNA-mediated cell pyroptosis is limited. For instance, there is a paucity of research on the efficacy of massage and miRNA-mediated cell pyroptosis. Additionally, the concrete mechanism and feasibility of targeting this pathway remain unclear. Concurrently, numerous contemporary rehabilitation techniques, such as transcranial direct current stimulation, are associated with the miRNA-mediated cell pyroptosis mechanism. Additionally, traditional techniques, such as Wuqinxi and the 8 Brocade of TCM, merit further investigation. It is hoped that this article will facilitate further investigation into the treatment of IS, thereby expanding the scope of clinical treatment for cerebral apoplexy.

**Table 1:** The related pathways and mechanisms of treatment of stroke through miRNA-mediated pyroptosis in traditional Chinese medicine.

Traditional Chinese medicine rehabilitation methods	Related miRNA		Activated by	Role of	References
Traditional Chinese medicine therapy	Traditional Chinese medicine	Ginsenoside Rd	miR-139-5p Fox O1 NLRP3 GSDMD	Inhibit the activation of NLRP3 inflammasome and Caspase-1, and to regulate their upstream related signaling pathways, reduce post-stroke pyroptosis and the related inflammation.	^[36]^
		Gastrodin	lnc RNA NEAT1/mi *R*-22-3p axis, IL-18, Caspase-1		^[37]^
	Prescription medicine	Taohong Siwu decoction	HMGB1/TLR4/NFκB and MAPK signaling pathways, NLRP3, GSDMD		^[38]^
Acupuncture therapy			Caspase-3, Caspase-8, and Caspase-9, Melatonin-NLRP3	The cortical cells were protected from ischemia induced apoptosis, and the percentage of cerebral infarction volume, the apoptosis rate of nerve cells and the activation level of microglia were significantly reduced.	^[39, 40]^
Tuina therapy			Caspase1, IL-1β, IL-18, GSDMD, NLRP3, miR-21, TLR4	Inhibit peripheral inflammation, regulate ion channels, inhibit the activation of glial cells, regulate brain function, and exert an analgesic effect therapy.	^[41]^
Traditional exercises therapy	Tai chi		TNF-α/Caspase8 pathway, BDNF, KLF2/HK1, NLRP3	Promote the production of neurotrophic factors or reduce vascular risk factors, increasing telomerase activity, oxidation, anti-apoptosis, neurotrophic and promote nerve regeneration.	^[47]^
	Mindfulness therapies including Qigong, Tai Chi, meditation and yoga		NF-κB		^[49]^
Modern rehabilitation technology	Repetitive transcranial magnetic stimulation		Caspase1, IL-1β, IL-18, ASC, GSDMD, NLRP1	Reduces the motor dysfunction and motor dysfunction caused by cerebral ischemia-reperfusion injury, inflammatory immune response and reducing pain.	^[51]^
	Hyperbaric oxygen treatment		lncRNA-H19/miR-423-5p/NLRP3 axis, Caspase-1, IL-1β, IL-18		^[52]^

BDNF = brain-derived neurotrophic factor, GSDMD = GasderminD protein, IS = ischemic stroke, lncRNA = long non-coding RNA, miRNA = microRNA, NLRP = nucleotide-binding oligomerization domain-like receptor protein.

## Author contributions

**Conceptualization:** Hanwen Ma.

**Data curation:** Luwen Zhu.

**Formal analysis:** Hanwen Ma.

**Investigation:** Hanwen Ma.

**Methodology:** Hanwen Ma.

**Project administration:** Hanwen Ma.

**Supervision:** Luwen Zhu.

**Validation:** Hanwen Ma, Luwen Zhu.

**Visualization:** Hanwen Ma, Luwen Zhu.

**Writing – original draft:** Hanwen Ma.

**Writing – review & editing:** Hanwen Ma, Luwen Zhu.
